# Direct and Delayed Mortality of *Ceriodaphnia dubia* and Rainbow Trout Following Time‐Varying Acute Exposures to Zinc

**DOI:** 10.1002/etc.5131

**Published:** 2021-07-20

**Authors:** Christopher A. Mebane, Christopher D. Ivey, Ning Wang, Jeffery A. Steevens, Danielle Cleveland, Michael C. Elias, James R. Justice, Kathryn Gallagher, Robert N. Brent

**Affiliations:** ^1^ Idaho Water Science Center US Geological Survey Boise Idaho; ^2^ Columbia Environmental Research Center US Geological Survey Columbia Missouri; ^3^ US Environmental Protection Agency Washington DC; ^4^ James Madison University Harrisonburg Virginia USA

**Keywords:** Metal toxicity, Toxicodynamics, Water quality criteria, Time‐variable toxicity, Latent effects, Pulse exposures

## Abstract

The potential for delayed mortality following short‐term episodic pollution events was evaluated by exposing cladocerans (*Ceriodaphnia dubia*) and rainbow trout (*Oncorhynchus mykiss*) to zinc (Zn) in various 1‐ to 48‐h and 1‐ to 96‐h exposures, respectively, followed by transferring the exposed organisms to clean water for up to 47 h for *C. dubia* and up to 95 h for trout for additional observation. For *C. dubia*, 1‐h exposures of up to 3790 µg Zn/L never resulted in mortality during the actual Zn exposures, but by 48 h, a 1‐h exposure to 114 µg/L, a concentration similar to the present US national water quality acute criterion for the test water conditions, ultimately killed 70% of *C. dubia*. With *C. dubia*, the speed of action of Zn toxicity was faster for intermediate concentrations than for the highest concentrations tested. For rainbow trout, pronounced delayed mortalities by 96 h only occurred following ≥8‐h exposures. For both species, ultimate mortalities from Zn exposures ≤8 h mostly presented as delayed mortalities, whereas for exposures ≥24 h, almost all ultimate mortalities presented during the actual exposure periods. With Zn, risks of delayed mortality following exposures to all concentrations tested were much greater for the more sensitive, small‐bodied invertebrate (*C. dubia*) than for the less sensitive, larger‐bodied fish (rainbow trout). These results, along with previous studies, show that delayed mortality is an important consideration in evaluating risks to aquatic organisms from brief, episodic exposures to some substances. *Environ Toxicol Chem* 2021;40:2484–2498. © 2021 The Authors. *Environmental Toxicology and Chemistry* published by Wiley Periodicals LLC on behalf of SETAC. This article has been contributed to by US Government employees and their work is in the public domain in the USA.

## Graphical abstract

Rainbow trout could withstand extreme Zn pulses of 1 to 3 h, with few subsequent deaths. Few *Ceriodaphnia dubia* died during 1‐ to 3‐h Zn pulses, but after 48 h, most were dead. Time to effects decreased as expected as rainbow trout were exposed to increasing Zn concentrations. Not so with *C. dubia*: time to effects was fastest in intermediate Zn exposures, with longer survivals in higher Zn exposures.

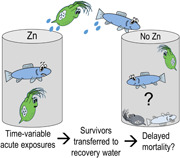

## INTRODUCTION

Water pollution sources are often episodic, which results in time‐varying concentrations of contaminants in receiving waters. Episodic pollution results from events and activities such as stormwater runoff from built‐up or disturbed areas including urban areas, highways, and industrial, agricultural, or mining operations. Brief pulse exposures of substances such as ammonia, sulfide, and metals can result from activities such as dredging and aquatic placement of dredged materials to maintain navigational channels. Capacity overloads or upsets from wastewater‐treatment plants and cleaning blowdowns of power plant cooling systems are among many other examples that can result in brief high pulses of pollutants (Seegert and Brooks 1978; Berthouex and Fan 1986; Fisher et al. 1994; Makepeace et al. 1995; Lee et al. 2004; Kayhanian et al. 2008; Brix et al. 2010; Corsi et al. 2010; Nimick et al. 2011; Scholz et al. 2011; Balistrieri et al. 2012).

To address the problem of time‐varying concentrations of pollutants in aquatic environments, the US Environmental Protection Agency (USEPA)'s national guidelines for deriving aquatic life criteria defined a 2‐number criteria framework expressing acute and chronic criteria in terms of concentration, duration, and exceedance frequency of allowable exposures (Stephan et al. 1985). Chronic criteria magnitudes (criterion continuous concentration [CCC]) were to be expressed as a 4‐d average concentration, and acute criteria (criterion maximum concentration [CMC]) were to be expressed as 1‐h average concentrations, with neither the CMC nor the CCC to be exceeded more than once every 3 yr. These durations were intended to address fluctuating concentrations in field conditions by limiting both the magnitude and duration of exceedances. Durations were consciously set to be shorter than the typical duration of acute and chronic toxicity tests to account for 1) delayed effects that may not be captured in standard toxicity tests, and 2) sensitive life stages that may only be briefly present during the course of a chronic toxicity test but for which effects might disproportionally occur. For example, Stephan et al. (1985) stated that for acute criteria, “one hour is probably an appropriate averaging time because high concentrations of some materials can cause death in one to three hours. Even when organisms do not die within the first hour or so, it is not known how many might have died due to delayed effects.”

Following the 1985 publication of the criteria derivation guidelines, all criteria documents that were written from 1985 until 2001 included a 1‐h averaging period for their acute criteria expressions. However, the 2001 acute cadmium (Cd) criterion set the acute averaging period to 24 h, as did the 2007 acute copper (Cu) criterion. No explanations were given in the criteria reports for the deviation from the guidelines (US Environmental Protection Agency 2001, 2007). Ammonia criteria updates in 2013 retained the 1‐h averaging period, on the rationale that ammonia was a fast‐acting acute toxicant (US Environmental Protection Agency 2013). When the acute Cd criterion was again updated in 2016, a 1‐h averaging period was again adopted on the basis that it was more appropriate in the absence of specific data supporting a change to a 24‐h averaging period (US Environmental Protection Agency 2016).

The purpose of the present study was to test a range of exposure durations including the 1‐ and 24‐h durations that have been used as acute averaging periods in aquatic criteria documents. The testing consisted of exposing organisms to zinc (Zn) in various combinations of durations and concentrations, followed by transfer of the exposed organisms to clean water for additional observations for delayed mortality. Zinc was chosen for testing because earlier work suggested that delayed mortality may be more of a concern for metals than organic compounds (Brent and Herricks 1998; Zhao and Newman 2004; Diamond et al. 2006; Erickson 2007). Moreover, in urban streams, Zn in stormwater runoff can rise from baseline concentrations of approximately 7 to 20 µg/L to >4 mg/L within minutes and can be toxic in short‐term exposures (Lee et al. 2004; Kayhanian et al. 2008). This urban baseline is approximately an order of magnitude higher than the global average background Zn concentration of approximately 0.6 µg/L in nonurbanized or industrialized river basins (Gaillardet et al. 2007). Our selection of Zn as a test material was further guided by the high solubility of Zn compounds, the relative ease of measurement of Zn in solution in the concentration ranges associated with acute toxicity, the relatively low risk to workers handling Zn in the study, and the relative lack of effect of minor changes in organic carbon concentrations on Zn toxicity to aquatic organisms.

## MATERIALS AND METHODS

### Test organisms

*Ceriodaphnia dubia* were obtained from the US Geological Survey's Columbia Environmental Research Center (CERC) in‐house cultures and mass‐cultured by placing 30 adults in each of eight 1‐L beakers with 900 mL of test water. The test water was prepared by diluting CERC well water (∼300 mg/L as CaCO_3_) with deionized water to result in a final hardness of 100 mg/L as CaCO_3_. Water was renewed on a Monday, Wednesday, Friday schedule by transferring the *C. dubia* to a clean 1‐L beaker with food and fresh test water. Every week, 4 of the beakers were restarted with <24‐h‐old neonates to ensure that adult *C. dubia* were readily available for testing. Adult *C. dubia* in each beaker were transferred to a new 1‐L beaker with food and fresh test water 24 h prior to the start of an exposure so that the <24‐h‐old young could be collected the next day and used for stocking exposures. The *C. dubia* were fed daily with 2 mL of a 3.0 × 10^7^ cells/mL concentration of *Raphidocelis subcapitata* (synonyms *Pseudokirchneriella subcapitata* and *Selenastrum capricornutum*; Aquatic BioSystems) and 2 mL of yeast, Cerophyl, trout chow (YCT; 1800 mg/L stock; Aquatic Biosystems).

The eggs (eyed stage) of rainbow trout (*Oncorhynchus mykiss*) were purchased from Trout Lodge/Hendrix Genetics and reared at CERC in control water. Rainbow trout eggs were held in an incubator consisting of a set of hatching trays with test water flowing through at 10 to 12 °C. Eggs and hatched fish were checked daily for mortality, and dead eggs and fish were removed. Swim‐up larval fish were transferred into a 500‐L flow‐through tank containing test water at 10 to 12 °C and fed newly hatched brine shrimp (Brine Shrimp Direct) and a salmon starter food (Rangen).

### Chemical analyses and water quality

A solution of the highest exposure concentration was first made by dissolving a known weight of ZnSO_4_ (99% purity; Sigma‐Aldrich) in a known volume of test water (control water). One‐half of the solution was then used to prepare the exposure solutions of lower exposure concentrations using 50% serial dilutions with the control water (Table 1). For the *C. dubia* exposures, the solutions were prepared 4 d prior to the start of the toxicity tests and held in a walk‐in cooler in the dark at 4 °C. On the day of the start of the tests, the solutions were removed from the walk‐in cooler and warmed up to 23 °C in a water bath for approximately 3 h. To ensure a full equilibration time, the test solutions for the later rainbow trout exposures were prepared 3 d before the start of the exposures and held at test temperature (12 °C) in a water bath before use for toxicity testing.

**Table 1:** General exposure/observation design for tests with *Ceriodaphnia dubia* and rainbow trout

Time exposed to Zn (h)	Total time observed (h)	Latency/recovery time (h)	Nominal exposure concentrations (µg Zn/L)	Zn chemical analysis sample (h)
*Ceriodaphnia dubia*				
1	48	47	0, 125, 250, 500, 1000, 2000, 4000	0
3	48	45	0, 125, 250, 500, 100, 2000, 4000	0
8	48	40	0, 63, 125, 250, 500, 1000, 2000	0, 8
24	48	24	0, 31, 63, 125, 250, 500, 1000	0, 24
48	96	48	0, 31, 63, 125, 250, 500	0, 22, 26, 48
*Oncorhynchus mykiss*				
1	96	95	0, 2000, 4000, 8000, 16 000, 32 000, 64 000	0
3	96	93	0, 2000, 4000, 8000, 16 000, 32 000, 64 000	0
8	96	88	0, 1000, 2000, 4000, 8000, 16 000, 32 000	0, 8
24	96	72	0, 500, 1000, 2000, 4000, 8000, 16 000	0, 24
96	144	48	0, 125, 250, 500, 1000, 2000	0, 44, 50, 96

Water temperature was monitored daily. Water quality characteristics (dissolved oxygen, pH, conductivity, hardness, alkalinity, and ammonia) were determined on composite water samples collected from the replicates in the control, medium, and high exposure concentrations at the beginning and the end of tests. Water samples for analyses of dissolved major ions and dissolved organic carbon (DOC) were collected from the control exposure chambers on day 1 for *C. dubia* and day 0 for rainbow trout. Water samples for Zn analyses were generally collected from each exposure concentration at 0, 8, 24, and 48 h during the *C. dubia* test where Zn exposure lasted 48 h and at 0, 8, 24, 48, and 96 h during the rainbow trout test where Zn exposure lasted 96 h. Treatments where Zn exposures were relatively shorter tracked similar water collection time points, with the latest collection points being limited by the duration of the individual Zn exposures (Table 1).

Two methods of analysis were used to measure dissolved Zn concentrations in the exposure chambers over time. In the first analytical method, colorimetric analyses of Zn were performed using a visible spectrophotometer (model HI801; Hanna Instrument) where Zn concentrations in filtered test water samples (filtered with 0.45 µm polyethersulfone [PES]) were determined using the zincon standard method (American Public Health Association, American Water Works Association, and Water Environment Federation 2005). Briefly, cyanide was used to complex Zn and other metals in solution, and cyclohexanone was then added to selectively release Zn from the cyanide complex. The newly liberated Zn ions complexed with the zincon indicator in a pH 9 buffered solution, causing a measurable color change at 620 nm that was proportional to the Zn ion concentration. The linear range of the spectrophotometer was 0 to 3.00 mg Zn/L, the baseline resolution was 0.01 mg Zn/L, and the limit of quantification (LOQ) was 0.03 mg Zn/L. Water samples were collected and measured by colorimetric analyses at the beginning and the end of all exposures (except at the end of the 2 short 1‐ and 3‐h exposures) and the end of the recovery times (Table 1). In the second analytical method, concentrations of dissolved Zn (0.45 µm PES) in test waters were measured using inductively coupled plasma‐mass spectrometry (ICP‐MS); samples for ICP‐MS analyses were collected at 24 h for *C. dubia* and at 0, 48, and 96 h for rainbow trout. The Zn samples for ICP‐MS analyses were preserved by acidification with high‐purity nitric acid, to result in a final acid concentration of 2% HNO_3_. Additional details on analytical methods and quality control results are given in the Supplemental Data.

### Toxicity tests

The Zn exposures used a static nonrenewal test with *C. dubia* for 1‐, 3‐, 8‐, 24‐, and 48‐h exposures and a static‐renewal test with rainbow trout for 1‐, 3‐, 8‐, 24‐, and 96‐h exposures in basic accordance with standard methods (US Environmental Protection Agency 2002a; ASTM International 2014). Test organisms were transferred to the control and different Zn exposure solutions for this series of pulsed exposures of different durations, using a matrix of concentrations and durations which were expected to produce partial toxicities in continuous exposures (Table 1). The anticipated target concentrations were based on preliminary testing as well as on the results of 24‐ and 48‐h Zn acute exposures with *C. dubia* (Ivey et al. 2017) and 24‐ to 96‐h Zn acute exposures with rainbow trout (Calfee et al. 2014), which had been conducted at CERC using similar test water. After the 48‐h exposure in water (i.e., water containing Zn treatments) for *C. dubia* and 96‐h exposure in water for rainbow trout, surviving organisms were placed into recovery water (i.e., control water), where they were monitored for recovery/delayed mortality for an additional 48 h, for total observation times of 96 and 144 h, respectively.

### C. dubia tests

Conditions for the 48‐h *C. dubia* test are summarized in Supplemental Data, Table S1. Test chambers were 50‐mL glass beakers with 40 mL test solution per beaker. There were 5 different exposure periods, each identified by the time the *C. dubia* were exposed to Zn (i.e., 1‐, 3‐, 8‐, 24‐, and 48‐h exposure). Each test of a given exposure period consisted of 6 or 7 treatments with varying Zn concentrations, and each treatment had 4 replicates (Table 1). Five organisms were impartially stocked per beaker into the exposure water. Within the 24‐h cohort used for stocking, unusually small and unusually large animals were excluded. All test chambers for a particular exposure were placed in a container and held in a water bath at 25 ± 1 °C. Each container had a lid to reduce evaporation. Test organisms were not fed, and exposure water was not renewed during the exposure and recovery times within the 48‐h test. At the end of 1‐, 3‐, 8‐, and 24‐h Zn exposure in the exposure water, mortality and immobilization (lack of movement except for minor spontaneous, random activity of appendages; ASTM International 2014) were determined for organisms in each beaker. Live organisms (including immobilized organisms) were transferred to clean water and observed for corresponding recovery times of 47, 45, 40, and 24 h (Table 1).

To minimize contaminant transfer to recovery water, organisms were briefly transferred to rinse water before being transferred to recovery water by pipette to a 250‐mL beaker of rinse water (i.e., control water) to dilute the contaminant before the transfer to recovery water, as described by Brent and Herricks (1999). Specifically, the organisms were drawn into a 1‐mL plastic pipette, and exposure water was decanted until the test organism was at the end of the pipette. The organism was then transferred to a new beaker with rinse water and the tip of the pipette placed just below the water surface, allowing the organism to swim out of the pipette and into the beaker. To control for possible handling stress, organisms in the controls were also transferred to new control beakers on the same schedule as the Zn‐exposed organisms in each of the 1‐, 3‐, 8‐, and 24‐h Zn exposures.

A conventional, continuous 48‐h exposure served as a positive control. At the end of the conventional acute 48‐h exposure, mortality was determined, and live organisms were briefly transferred to rinse water and then recovery water and observed for an additional 48 h to determine delayed effects. In addition to the mortality determination at the end of the 1‐, 3‐, 8‐, 24‐, and 48‐h exposures, mortality was determined at 6, 12, 16, and 36 h. For the conventional 48‐h duration treatment only, following transfer to recovery water, surviving *C. dubia* were fed 0.2 mL of the YCT and 0.2 mL of the algal concentrate (described in the section *Test organisms*) per beaker once daily after transfer and after the water renewal for controls at 48 and 72 h (ASTM International 2013). Recovery water renewal was done by transferring organisms to new beakers with new recovery water.

### Rainbow trout tests

Conditions for the 96‐h rainbow trout test are summarized in Supplemental Data, Table S2. Test chambers were 3800‐mL glass jars with 3000 mL test solution per jar. Five fish (~20 d post‐swim‐up; average individual wet wt 0.38 g based on a pooled sample of 10 fish) were impartially stocked per jar. Each treatment had 4 replicates. The mean loading in each jar was 0.63 g/L wet weight, which met the ASTM International (2014) recommendation of <0.8 g of organism/L for temperatures at or below 17 °C. Jars were held in a water bath at 12 ± 1 °C, and the fish were not fed in all exposures of up to 96 h. Approximately 80% of the water in a jar during the exposure or recovery time, depending on the duration treatment, was renewed at 48 h into the total 96‐h observation time. At the end of 1‐, 3‐, 8‐, 24‐, and 96‐h exposures, mortality (no respiratory movement) and immobilization (lying down on their side and lack of movement) in each replicate were determined; and live fish (including immobilized fish) were briefly transferred to rinse water before being transferred to recovery water for corresponding recovery times of 95, 93, 88, and 72 h (Table 1). Specifically, the fish were transferred by a net to a 30‐L container of rinse water (i.e., control water) before transfer to recovery water. The conventional continuous 96‐h exposure served as a positive control. In addition to the mortality determination at the end of the 1‐, 3‐, 8‐, 24‐, and 96‐h exposures, mortality was determined at 6, 12, 16, 36, 48, and 72 h.

At the end of the conventional acute 96‐h exposure, mortality was determined, and live organisms (including immobilized fish) were briefly transferred to rinse water before being transferred to recovery water and observed for an additional 48‐h period to determine any delayed effects. The fish were fed <24‐h‐old brine shrimp nauplii 2 times daily at an interval of 6 h during the 48‐h observation period. Sufficient numbers of nauplii were provided to assure that some nauplii remained alive in test jars for several hours after each feeding (US Environmental Protection Agency 2002b). After 24 h, uneaten brine shrimp and other debris were removed by siphoning with a small‐bore glass tube, and approximately 80% of the recovery water was renewed before the first feeding of the day.

### Data analysis

Effect concentration percentile estimates (e.g., concentration causing 50% or other percentile effect [EC50]) were determined using the Toxicity Relationship Analysis Program (TRAP; Erickson 2015). Calculations were conducted for each exposure and observation time. Within the TRAP software, the data were log10‐transformed and modeled using a 3‐parameter fit with normal (Gaussian) distribution using the tolerance distribution procedure. Confidence intervals were estimated at the α = 0.05 level. This procedure was repeated for each exposure. If the data did not meet the requirements of the 3‐parameter tolerance distribution model (i.e., at least 2 partial responses) but had bracketing responses with either a no‐observed‐effect concentration or a lowest‐observed‐effect concentration with >50% survival and complete mortality at the next higher treatment, EC50s were estimated by interpolating between these 2 bracketing concentrations, and the bracketing concentrations were considered the confidence interval.

Median time to immobilization or death (ET50s) were similarly calculated for tests with observed responses at multiple times from the same treatments with similar concentrations. The same models were used within the TRAP software, substituting time for concentration.

## RESULTS

### Chemical analyses and water quality

All measured water quality parameters for the *C. dubia* and rainbow trout tests were similar within each treatment and across different treatments (Supplemental Data, Table S3): dissolved oxygen concentrations averaged 8.9 mg/L with a standard deviation (SD) of 0.7 (*n* = 71); pH averaged 7.84 (SD 0.29, *n* = 71), alkalinity averaged 92 mg/L as CaCO_3_ (SD 4.8, *n* = 71), hardness averaged 104 mg/L as CaCO_3_ (SD 4.8, *n* = 71), and ammonia averaged 0.128 mg N/L (SD 0.285, *n* = 70) (Supplemental Data, Table S3). Major ion concentrations were also similar across tests (Supplemental Data, Table S4): Ca ranged from 25 to 27 mg/L, Mg from 8.4 to 8.6 mg/L, K from 0.9 to 1.0 mg/L, Na from 8.3 to 9.2 mg/L, Cl from 10 to 11 mg/L, and SO_4_ from 17 to 19 mg/L. The concentration of DOC was 0.4 mg/L for the tests with both species. More details on all data generated in the present study are available from a data repository (Ivey and Mebane 2019) and in the Supplemental Data of the present study.

Zinc concentrations measured by ICP‐MS ranged from 78 to 100% of nominal for the *C. dubia* exposures and from 60 to 110% of nominal for the rainbow trout exposures (Supplemental Data, Table S5). The lower measured concentrations of Zn relative to nominal concentrations (<80%) were all from Zn exposures >4000 µg/L (Supplemental Data, Table S5). Although the relatively high (~100 µg/L) LOQ of the colorimetric method makes it unsuitable as a stand‐alone analytical method, the colorimetric analyses allowed us to analyze an increased number of samples at more time points at a much lower cost and to obtain results on the same day. For *C. dubia* exposures, Zn measured colorimetrically for the nominal exposures ≥125 µg/L ranged from 89 to 104% of the ICP‐MS concentrations (Supplemental Data, Table S5). Concentrations of Zn measured colorimetrically during the rainbow trout exposures (which had greater nominal Zn concentrations than the *C. dubia* exposures) ranged from 87 to 135% of the ICP‐MS for the nominal exposures ≥125 µg/L, with values >100% all from very high concentrations (>6000 µg/L; Supplemental Data, Table S5). The frequent colorimetric analyses also allowed testing of the stability of exposure concentrations. For the 5 *C. dubia* tests with at least 3 colorimetric measurements each (i.e., not those exposures abbreviated by complete mortalities), the exposure coefficients of variability (*CV*) ranged from 6 to 18%. Likewise, with rainbow trout, *CV*s ranged from 5 to 16% (Supplemental Data, Table S5). The *CV* results suggested that the Zn exposure concentrations were stable over the exposure periods; therefore, only Zn concentrations measured by ICP‐MS were used for effect concentration calculations.

### C. dubia test

The study‐wide average control survival of *C. dubia* at the end of the combined 48‐h exposure and recovery periods was 90%. Control survival in the 1‐, 24‐, and 48‐h *C. dubia* tests was 100%, whereas the control survival in the 3‐ and 8‐h tests were 80 and 75%, respectively. Each *C. dubia* test had 4 control replicates, and of the 20 control replicates across the 5 tests, 17 had 100% survival, 1 had 80% survival, 1 had 20% survival, and 1 had 0% survival. In both tests in which a single control replicate failed (the 3‐ and 8‐h Zn exposures), the high mortalities all occurred in a single replicate, with 0% mortalities in 3 of the 4 replicates and 80 and 100% mortality in a single replicate (Supplemental Data, SI‐6). The causes of the high mortalities are unexplained; they began shortly after capture and transfer of the organisms to “recovery” control exposures in these individual replicates. A standard guide for conducting an acute toxicity test following standard methods recommends that exposures should usually be considered unacceptable if >10% of the organisms in the controls die during the test (ASTM International 2014). However, these were not standard tests in that control animals were captured and transferred to fresh control water on the same schedule as the Zn‐exposed organisms, and thus were subjected to much more handling than in a standard test. Because 100% of the Zn‐exposed *C. dubia* ultimately died in all of the treatments in both of the 3‐ and 8‐h exposures, the 20 and 25% control mortalities, respectively, do not affect our interpretations of those tests.

**Figure 1:**
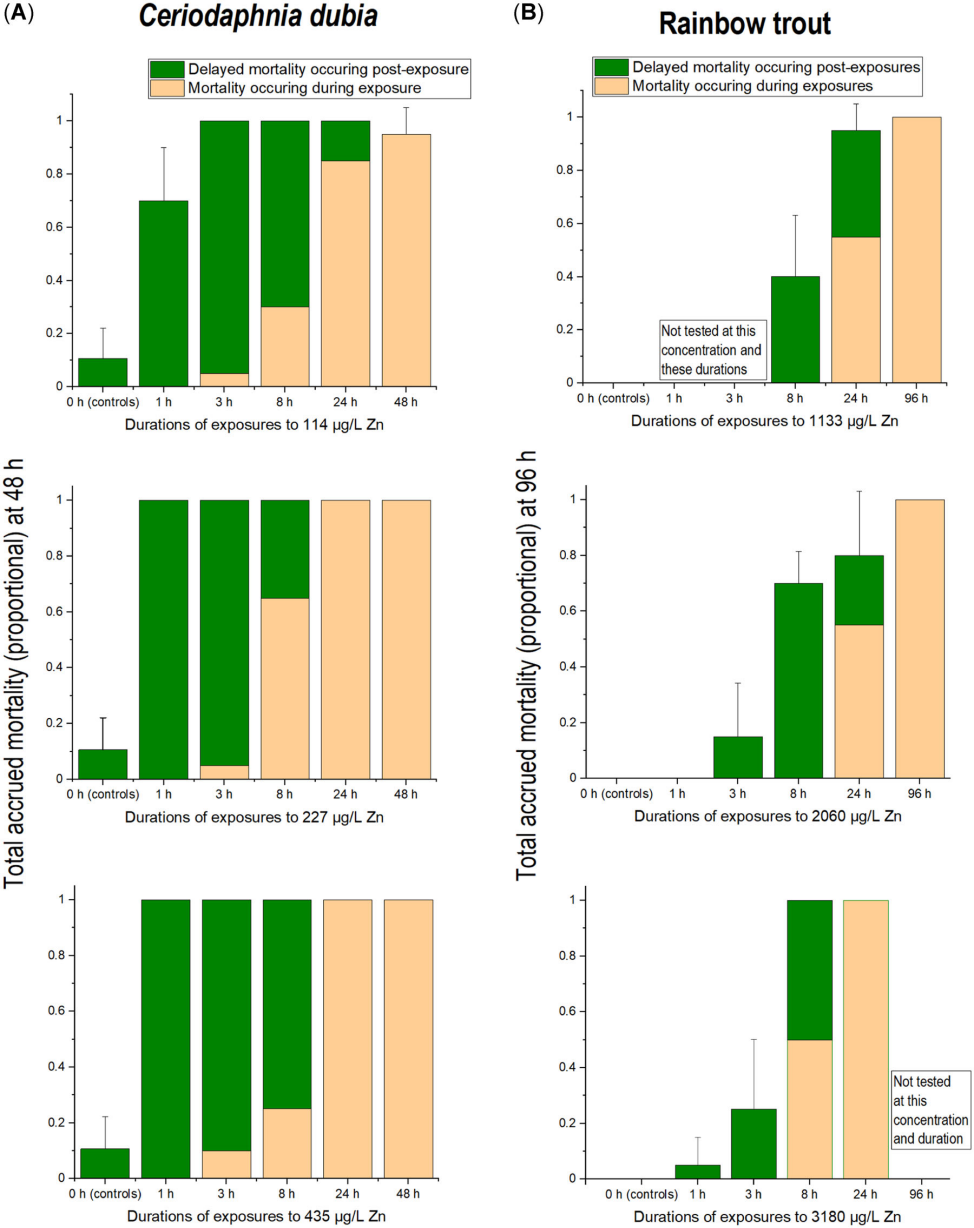
Summary of mortalities occurring during exposures to Zn at increasing durations and total delayed mortalities that continued to accrue after the organisms were transferred to clean water until the end of the observation periods at 48 or 96 h, respectively, for *Ceriodaphnia dubia* and rainbow trout. Mortalities are expressed as proportions; control mortalities at 48 or 96 h are the grand averages for the experiment. Error bars show standard deviations of total mortalities.

The *C. dubia* EC50s for the 5 exposure periods at each time point are shown in Table 2. In general, the *C. dubia* EC50s decreased as exposure times increased (Table 2). For the conventional acute 48‐h toxicity test, the Zn EC50 was approximately 37 µg/L. The *C. dubia* 1‐ and 3‐h exposures did not exhibit consistent mortality greater than that of controls until 24 or more hours after the exposure (Figure 1A). The 8‐, 24‐, and 48‐h exposures resulted in similar EC50 values by 48 h, with no latent effects observed in the 48‐h exposures (Figure 1).

**Table 2:** Median effect concentrations (EC50s µg Zn/L) in tests with cladocerans (*Ceriodaphnia dubia*) and rainbow trout (*Oncorhynchus mykiss*) over different exposure periods^a^

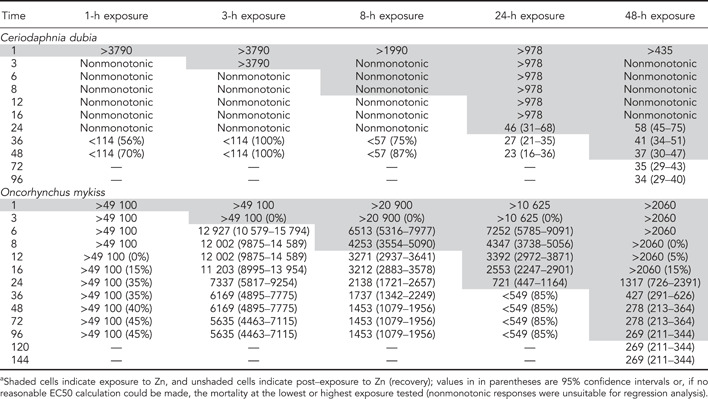

### Rainbow trout test

The control survival for all the rainbow trout exposure and recovery times was 100%. All rainbow trout EC50s for the 5 exposure periods at each time point are given in Table 2. As observed in the exposures with *C. dubia*, EC50s from the trout exposures decreased as exposure times increased (Table 2).

**Figure 2:**
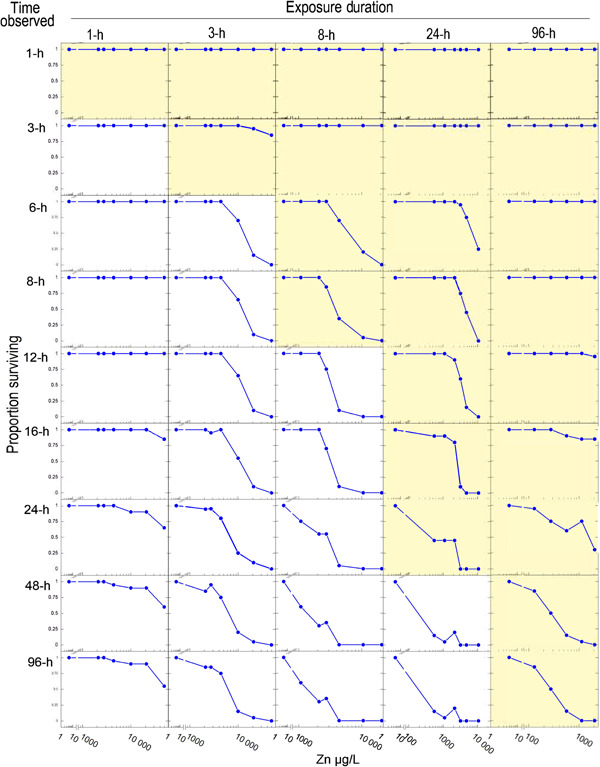
Progressive mortality of rainbow trout at different observation times, exposure times, and Zn concentrations. Shaded graphs show survival during Zn exposures; unshaded graphs show survival following Zn exposures. Observations at 36 and 72 h are omitted for space. Unlike *Ceriodaphnia dubia*, rainbow trout mortalities progressed with increasing exposure and time, with few irregularities or reversals.

For rainbow trout exposed to Zn for 8 and 24 h, there were pronounced delayed mortalities observed by the end of the 96‐h observation period, with most of the mortality actually occurring in the first 16 to 12 h of the postexposure recovery period, respectively (Figure 1B). That is, by 36 h into the entire observation period, mortalities largely ceased; fish that survived to 36 h mostly survived to the end of the exposures at 96 or 144 h. Only environmentally exorbitant Zn exposures ≥10 000 µg/L killed rainbow trout following a 1‐h Zn exposure (Figure 2). Even when exposures were extended to 3 h, >4000 µg/L Zn was required to produce mortalities by 96 h into the total observation period (Table 2).

### Time to death

With *C. dubia*, for those treatments producing >50% effects, median times to immobilization or death ranged from 4.4 to 48 h (Table 3). The fastest times to effects were at the intermediate treatment of 227 µg/L, regardless of exposure and recovery times. Both lower and higher exposure concentrations produced significantly longer times to effects.

**Table 3:** Median time to death or immobilization in tests with cladocerans (*Ceriodaphnia dubia*) and rainbow trout (*Oncorhynchus mykiss*) for different exposure concentrations and exposure times^a^

Average exposure (µg/L Zn)	ET50 (h) following 1‐h exposure	ET50 (h) following 3‐h exposure	ET50 (h), 8‐h exposure	ET50 (h), 24‐h exposure	ET50 (h), 48‐h exposure
*Ceriodaphnia dubia*				
24	nt	nt	nt	48 (32–73)	>48
57	nt	nt	8.3 (4.8–14)	15 (12–17.9)	22 (19–26)
114	31 (20–50)	21 (18–24)	12 (9.7–16)	18 (15.9–20)	4.4 (1.6–12)
227	12 (9.0–15)	20 (18–24)	6.0 (3.8–9.3)	17 (15–18)	4.5 (2.8–7.2)
435	18 (15–21)	20 (17–24)	24 (16–36)	17 (16–24)^b^	17 (16–20)
978	26 (24–36)^b^	21 (19–23)	20 (17–24)	20 (18–22)	nt
1990	26 (24–36)^b^	26 (23–28)	14 (12–17)	nt	nt
3790	27 (24–36)^b^	22 (20–25)	nt	nt	nt
*Oncorhynchus mykiss*				
130	nt	nt	nt	nt	>96
276	nt	nt	nt	nt	65 (49–86)
569	nt	nt	nt	30 (25–35)	28 (25–32)
1133	nt	nt	>96	25 (22–29)	29 (26–34)
2060	>96	>96	41 (34–50)	29 (24–35)	6.9 (6–8)^b^
3180	>96	>96	38 (28–50)	11 (9.9–13)	nt
4760	>96	>96	7.5 (6.4–8.8)	7.7 (6.8–8.8)	nt
10 625	>96	16 (12–22)	5.1 (4.4–6.0)	5.7 (3–6)^b^	nt
20 900	>96	4.7 (3–6)^b^	4.2 (3–6)^b^	nt	nt
49 100	>96	3.3 (3–6)^b^	nt	nt	nt

^a^ Insufficient partial responses to calculate reasonable statistical confidence limits; the bracketing time periods with low and high responses are considered lower and upper confidence limits.

^b^ Values in parentheses are 95% confidence intervals.

ET50 = median time to immobilization or death; nt = not tested.

With rainbow trout, median times to immobilization or death ranged from 3.3 to 65 h. The speed of effects increased steadily with increasing exposure concentrations, regardless of exposure and recovery times (Table 3).

## DISCUSSION

With Zn, risks of delayed mortality following brief exposures to all concentrations tested were much greater for a more sensitive, small‐bodied invertebrate (*C. dubia*) than for a less sensitive, larger‐bodied fish (rainbow trout). For both species, ultimate mortalities from Zn exposures ≤8 h in duration mostly presented as delayed mortalities during the recovery periods, whereas for exposures ≥24 h, almost all mortalities ultimately presented during the actual exposure periods (Figure 1). The importance of considering the risk of potential delayed mortality following episodic exposures was most apparent with the 1‐ and 3‐h exposures of *C. dubia*. For example, in a set of 3‐h exposures at lower, intermediate, and higher Zn concentrations, only 5 to 10% mortality occurred within that 3‐h exposure period, yet 100% eventually died. In the 1‐h exposures, no mortalities occurred during the actual exposures, yet 70 to 100% ultimately died (Figure 1). Compared as ratios, the 1‐h *C. dubia* EC50 was >3760 µg/L, but the 1‐h delayed mortality at 48 h was approximately 100 µg/L, or approximately 40 times lower. With rainbow trout, these differences were much less. For example, the rainbow trout EC50 for an 8‐h exposure was 4250 µg/L Zn; but when the trout were transferred to clean water and observed out to 96 h, the 96‐h median lethal concentration (LC50) was approximately 3 times lower, at 1450 µg/L.

The nominal 125 µg/L Zn exposure (114 µg/L average measured) was close to the acute aquatic life criterion for Zn (120 µg/L) at the average hardness of 103 mg/L as CaCO_3_ for that exposure series (US Environmental Protection Agency 1996). The observation that a 1‐h exposure at the criterion concentration resulted in 70% mortality to a sensitive invertebrate by 48 h and that a 3‐h exposure killed 100% by 36 h indicates that brief episodic pollution events of moderate magnitude could harm at least some sensitive, small‐bodied invertebrate taxa.

### Progression of effects of Zn over time

In the rainbow trout tests, mortalities accrued in mostly predictable patterns, increasing with higher exposure concentrations, with deaths increasing with exposure times until they largely ceased at approximately 36 h after initiation of exposures. These patterns were qualitatively similar in both the continuously exposed fish and the fish that had been transferred to recovery water (Figure 2).

**Figure 3:**
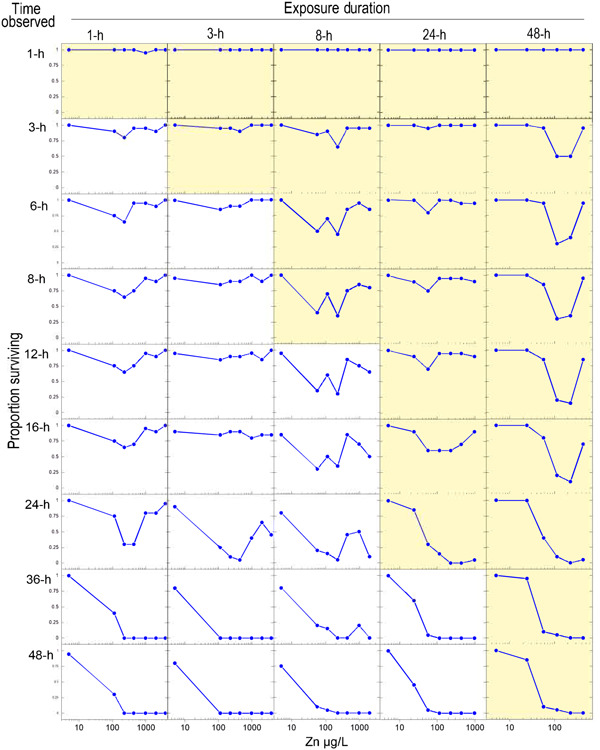
Progressive mortality of *Ceriodaphnia dubia* at different observation times, exposure times, and Zn concentrations. Shaded graphs show survival during Zn exposures; unshaded graphs show survival following Zn exposures. Speed of action was not fastest in the highest Zn concentrations, but rather, the onset of mortalities was fastest in the intermediate concentrations. The intermediate concentrations consistently had more severe effects at approximately 12 to 24 h than did the high concentrations, regardless of whether the organisms were subject to ongoing Zn exposure or not.

In the *C. dubia* exposures, the speed of action of Zn toxicity was faster at intermediate concentrations than it was at higher concentrations. This can be seen in Figure 3, where at 1 h survival is nearly 100% in all exposures. By 3 to 6 h, mortalities are evident across all 5 tests in the intermediate exposures (~100–300 µg/L) but not in the higher exposures. This pattern held until approximately 24 to 36 h, by which time survival dropped to near zero in all of the intermediate and high exposures. By 36 h, the survival patterns in all 5 tests had collapsed into classic log‐normal concentration–response curves, except for those with complete mortalities in all treatments.

Because the 5 tests of different exposure times were independent of each other and the patterns were observed across the tests, we are confident that this faster speed of action at intermediate concentrations is a phenomenon, rather than a data problem. We are unaware of previous reports of this phenomenon but surmise that because the phenomenon was gone by 48 h, it would be highly unlikely to be detected in standard acute testing.

The mechanism for intermediate concentrations killing faster than higher concentrations of a toxicant is unexplained. Particularly puzzling to us is that the brief (1‐ and 3‐h) exposures also produced this progression of effects, with severely increased mortalities appearing by 20+ h after the exposures ended. Plausible factors suggested by the patterns include that the high concentrations triggered a defensive mechanism that delayed toxicity and that the high concentrations caused an initial inhibition of a biological function(s) that differed from the effects that ultimately killed them. In fish, acute Zn toxicity results from hypocalcemia as Zn competitively inhibits Ca uptake across gill epithelial cells via the epithelial Ca channel. When rainbow trout have been transferred to high‐Zn water, rapid decreases in the affinity of Zn for branchial influx and reduced Zn uptake have been observed (Hogstrand 2011). Acute toxicity in *Daphnia* has been shown to result from rapid accumulation of Zn, when whole‐body Zn accumulation passed a threshold of approximately 3.5 times background (Wang and Guan 2010). Thus, it seems plausible that physiological changes reducing Zn uptake at high concentrations resulted in slower accumulation to lethal levels. Higher accumulations of Zn in intermediate exposures than in higher exposures have also been noted in acute exposures of fish (McRae et al. 2016) and in longer‐term exposures of insects to Zn and other metals (Tomczyk et al. 2018; Mebane et al. 2020b), which suggests multiple factors in play. In positing these possible factors, we are assuming that these homeostasis and toxicity mechanisms are similar across freshwater animals (Mebane et al. 2020a). An additional possible factor is that filtration rates in *Daphnia* have been observed to decline with metal exposure (Muyssen et al. 2006; Bownik 2020). Filtration by the thoracic appendages has an important role in ventilation because their activity provides a constant water flow, providing oxygen to the organism (Bownik 2020). While this phenomenon of faster effects at intermediate concentrations is intriguing and warrants further inquiry than our speculations in the present study, the results suggest that it may not necessarily be important in regulatory 48‐h toxicity testing because by 36 h survival had largely stabilized into classic sigmoid concentration–response patterns (Figure 3).

### Comparisons to earlier work

The sensitivities to Zn of both the rainbow trout and the *C. dubia* in the present study were within the ranges of previous testing in our laboratory and lower than average values compiled for both species. Two previous 48‐h Zn exposures with *C. dubia* under similar test conditions yielded EC50 estimates of 136 and <83 µg/L (i.e., complete mortality occurred at the lowest concentration tested, 83 µg/L; Ivey et al. 2017). The present 48‐h EC50 of 37 µg/L was similar to the previous <83 µg/L result in that the 2 exposures in the present study that bracketed 83, 57, and 114 µg/L had 90 and 95% mortalities, respectively. In a compilation of 15 hardness‐adjusted *C. dubia* tests (hardness 85 mg/L, similar to the test hardness of ~100 mg/L), EC50s ranged from 46 to 1188 µg/L, with a species geometric mean of 263 µg/L (DeForest and Van Genderen 2012). Variability in EC50s was likely influenced by toxicity‐modifying factors other than hardness such as pH, DOC, and organism age.

With rainbow trout, the conventional acute 96‐h toxicity test EC50 was 269 µg Zn/L, which is similar to the 96‐h EC50 of 253 µg Zn/L obtained with rainbow trout by Calfee et al. (2014) under similar test conditions with similar‐aged fish. In a compilation of 56 hardness‐adjusted rainbow trout tests, EC50s ranged from 122 to 2383, with a species geometric mean of 690 µg/L (DeForest and Van Genderen 2012). Likewise, with *C. dubia*, variability in EC50s was likely influenced by toxicity‐modifying factors other than hardness, such as pH, DOC, and organism age or developmental stage.

**Figure 4:**
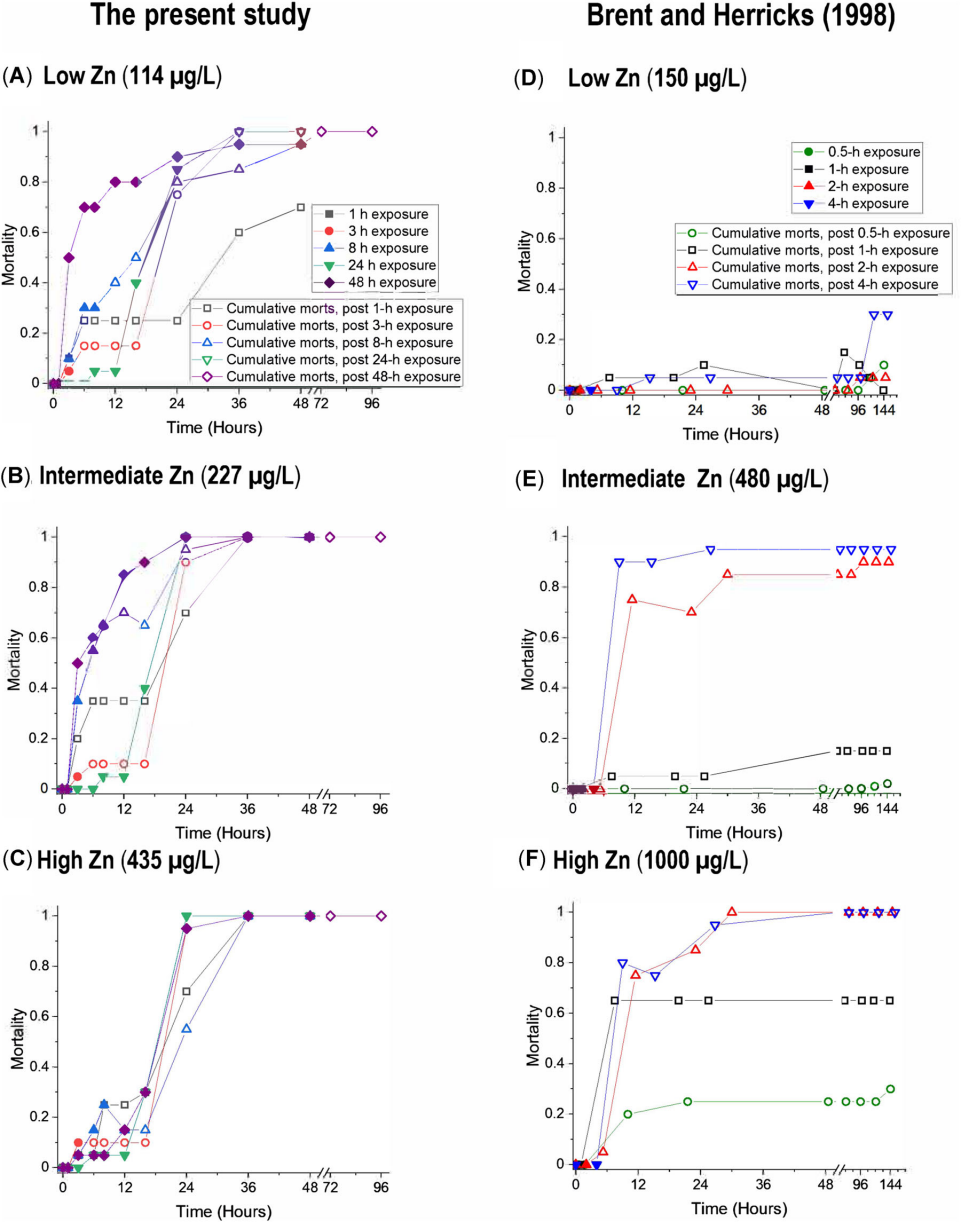
Comparison of cumulative mortalities to *Ceriodaphnia dubia* during and after exposures to lower, intermediate, and higher Zn concentrations for different durations during the present study and the study by Brent and Herricks (1998). Mortalities that occurred during the actual Zn exposures are plotted with solid symbols; delayed mortalities occurring after transfer to clean water are plotted as open symbols. morts = mortalities.

The pulse exposure tests reported in the present study followed a similar design as was used by Brent and Herricks (1998) with *C. dubia*, amphipods (*Hyalella azteca*), and fathead minnow (*Pimephales promelas*). The organisms were briefly exposed to varying concentrations of Cd, Zn, or phenol for up to 4 h and then transferred to recovery water and observed out to 144 h. With Cd and Zn, exposures of 30 min and longer produced postexposure mortalities. With *C. dubia*, a set of lower, intermediate, and higher Zn exposures are contrasted between their study and the present study (Figure 4). Although the exposures were shorter in the 1998 study (0.5–4 h) than in the present study (1–48 h), the overall patterns between the 2 studies were strikingly similar. Few to no mortalities (as immobilization) occurred in the first 1 to 4 h, yet high mortalities accrued by 24 h. The *C. dubia* in the present study were more sensitive to Zn than those in the earlier study, as shown by high delayed mortality in the 1‐ and 3‐h exposures to 114 µg/L Zn in the present study but little mortality from 1‐ and 4‐h exposures to 150 µg/L Zn in Brent and Herricks (1998). Some of the differences in Zn sensitivity between the 2 studies were presumably influenced by water hardness differences (100 vs 170 mg/L as CaCO_3_ in the present and earlier study, respectively). Using the ln(hardness) versus ln(EC50) hardness–toxicity regression slope of 0.8473 from the US Environmental Protection Agency (1996) to normalize the earlier testing at hardness of 170 to 100 mg/L, the hardness‐normalized exposures were lower, with 150‐µg/L “low” exposure in the earlier study more like a 96‐µg/L exposure in the present study's water, the 480‐µg/L “intermediate” exposure more like a 306‐µg/L exposure in the present water, and the “high” 1000‐µg/L exposure at hardness 170 mg/L more like a 638‐µg/L exposure at hardness 100 mg/L (Figure 4).

In a comparison of subacute responses of *Daphnia magna* and fathead minnow to 24‐h pulse exposures of ammonia, Cu, and Zn, only Zn exposures resulted in delayed mortality (Diamond et al. 2006). Unlike the present study, 3‐ to 6‐h Zn pulses had no effect on *D. magna* survival or reproduction. Diamond et al. (2006) attributed the delayed effects of Zn to slower uptake or depuration relative to other tested substances.

Immobilization accruals were not always strictly monotonic with increasing time or exposure, which is consistent with previous work showing a strong stochastic element to toxicity and recovery (Zhao and Newman 2007). Both the present study and the Brent and Herricks (1998) study also had some temporary recovery of immobilized *C. dubia*, which can be seen as decreases in cumulative mortalities over time in some exposures (e.g., Figure 4B, the 8‐h exposure and Figure 4E, the 2‐h exposure). However, these zombie *C. dubia* were unable to fully recover, as indicated by increasing mortalities recorded at the next observation times, even though the animals experienced no further Zn exposures.

### Implications for modeling toxicity from episodic exposures

Approaches to assess risk from the episodic nature of exposure to pollutants can be grouped into experimental approaches such as that of the present study and modeling approaches (reviewed in Gordon et al. 2012). The present results indicate that modeling approaches to predict effects of episodic pollution events using conventional toxicity test data can be severely misleading. For example, Mancini (1983) devised an approach for determining the time period for episodic pollution effects to be considered independent (nonadditive) between events. If mortality of aquatic organisms was presumed to result from contaminant accumulation in the bodies reaching a critical threshold, then data from classical bioassay tests obtained using constant toxicant exposure concentrations could be used to infer uptake and depuration rates. His approach was rearranged by Erickson (2007) to enable calculation of the speed of action of acute toxicity of chemicals. Erickson (2007) also illustrated how delayed mortality could be incorporated into speed of action modeling. However, when calculating time‐varying LC50s from conventional toxicity test data, there is no way to tell which part of the exposures caused effects. In other words, even if a researcher observed and reported cumulative toxicity frequently, there is no way to differentiate whether a no‐effect observation at 4 h, for example, truly reflects no effects or whether the exposure triggered delayed effects that might not be observed until 24 h and would be mistallied among the 24‐h effects.

### Implications for biotic ligand and related mechanistic models

The present results also have implications for biotic ligand models (BLMs) for metal toxicity. Such models were developed using experiments that showed metal accumulation on gill surfaces of rainbow trout following short‐term (1‐ to 3‐h) exposures that predicted later toxicity at 96 h (Playle 1998; Alsop and Wood 2000; Todd et al. 2009; Mebane et al. 2020a). The present results with rainbow trout showed very little toxicity following 1‐ to 3‐h exposures and indicate that these “lethal accumulation” values are correlates of eventual toxicity but are not necessarily causative of toxicity. The contrasting *C. dubia* results for which brief (1‐ to 3‐h) exposures did result in later toxicity suggest biodynamic differences in which the smaller organism has faster uptake and faster and more severe effects (Grosell et al. 2002; Gergs et al. 2015).

The brief (1‐ to 3‐h) exposures only produced rainbow trout mortalities following extremely high Zn concentrations (>4000 µg/L). This suggests that kinetic limitations of Zn gill accumulation could have prevented toxicity from very brief exposures. Alsop and Wood (2000) found that exposure of rainbow trout to 1000 µg/L Zn for 1.25 h resulted in similar gill accumulation as did exposure of 200 µg/L for 7.75 h, with the accumulation curves flattening by approximately 1000 µg/L Zn. Those results indicate that there may be little dose response for short‐term exposures (Alsop and Wood 2000). In in situ toxicity tests in a stream subject to strong diel swings in Zn and pH, the toxicity of cutthroat trout (*Oncorhynchus clarkii*) matched the average exposure periods better than peak exposures (Balistrieri et al. 2012). These factors are congruent with our observation that only extremely high dissolved Zn concentrations killed fish after a 1‐h exposure, when followed by 95 h postexposure in Zn‐free water.

Aquatic organisms can also depurate Zn, and toxicity is thought to result when accumulation exceeds clearance mechanisms and damage exceeds repair mechanisms, resulting in damage to a critical organ or critical functions (Butcher et al. 2006). Just what critical organs or functions are likely to be damaged by short‐term Zn exposures is unclear. With Zn (as well as some other metals), while toxicity is related to interference with internal hydromineral balances, the specific mechanisms remain uncertain. In early detailed studies of physiologic changes in rainbow trout associated with acute Zn exposures, Spry and Wood (1984) exposed fish to twice their acute LC50 concentration and observed rapid lethality. No fish died before 6 h of exposure, but the number of dead trout rose rapidly after 9 h, with complete mortality by 15 h. The mortalities were accompanied by acid–base responses of rainbow trout, consistent with death from hypoxia caused by severe gill damage from an external action of Zn and a 25% decline in plasma Ca levels (Spry and Wood 1984). Yet, further experimental approaches that changed variables such as exposure duration or concentration, size of fish, or calcium levels in exposure water or diet have produced differing physiological changes associated with gross effects such as mortality, growth, and swimming performance (Spry and Wood 1985; Hogstrand et al. 1995; Alsop and Wood 2011; Hogstrand 2011). These diverse and sometimes contradictory reports make it difficult to relate the gross empirical results (i.e., mortality patterns) obtained in the present study to the mechanisms of Zn toxicity to fish that may either trigger an irreversible physiological cascade of effects leading to death or produce no obvious lasting harm.

With daphnids and other invertebrates, we are aware of far less mechanistic toxicity work with Zn than with fish. Muyssen and Janssen (2002) found that uptake and depuration with a cladoceran (*D. magna*) was rapid, with major increases and decreases occurring within 24 h. Despite this, the severe delayed effects with cladocerans in the present study indicate that our understanding of Zn mechanisms of toxicity is incomplete. In a study with amphipods (*H. azteca*), Zn uptake was faster than depuration, although both processes were slower than in *D. magna*; maximum uptake in *H. azteca* occurred by 3.5 d (Borgmann and Norwood 1995). Among insects, toxicity of Zn and other metals is strongly related to accumulation dynamics, and stronger accumulators with higher uptake rates are generally more sensitive (Poteat and Buchwalter 2014).

Nevertheless, even when the true toxicokinetic functions are not known, empirically based modeling approaches that combine the metal bioavailability predictions of BLMs with biodynamic calculations have had some success in relating time‐variable external and internal metals concentrations to effects (Meyer et al. 2007; Electric Power Research Institute 2008; Veltman et al. 2010; Gordon et al. 2012; Balistrieri et al. 2020). While the understanding of mechanisms is incomplete, these studies suggest mechanisms and avenues of inquiry to better understand which organisms could be expected to be more vulnerable to pulsed metal exposures.

### Design considerations for testing differing exposure times

Our final observation and recommendations relate to the difficulty of addressing lethality over a wide range of times. Despite conducting pilot testing to focus test exposures and a test design with 7 treatments and 20‐ to 30‐fold ranges in exposures, we often obtained semiquantitative less‐than or greater‐than results. In future testing of time‐varying exposures, we suggest emphasizing testing of more concentrations than would be necessary for conventional exposures for fixed time periods, with a possible trade‐off of less emphasis on replication.

Test results with *C. dubia*, and probably all daphnids, can be highly variable. In the present study, the 48‐h EC50 of approximately 37 µg/L was as much as 3 times lower than those obtained in earlier testing in the same laboratory, with animals from the same culture and the same culture and test water sources. In this earlier testing (Ivey et al. 2017), 2 tests with *C. dubia* and Zn were conducted. One LC50 (<83 µg/L) in which complete mortality occurred at the lowest concentration tested was not necessarily dissimilar to the present 48‐h EC50 of 37 µg/L but was 3 times lower than the repeated test with an EC50 of 136 µg/L (Ivey et al. 2017). In DeForest and Van Genderen's (2012) USEPA‐style compilation and normalization of Zn, freshwater toxicity data showed a 10 times variability of *C. dubia* acute LC50s following BLM normalization for differing influences of water chemistry. Tests of the same clone in the same study varied by up to 3 times (DeForest and Van Genderen 2012). With *D. magna*, acute Zn LC50s ranged over a factor of 10 times when exposed in the same test water (Muyssen et al. 2005); and in a compilation of Cd test results, *D. magna* LC50s ranged over 100 times, even after hardness–toxicity adjustment (Mebane 2006). A likely contributing factor to this variability is the age of the organisms at test initiation. The test protocol for testing the acute toxicity of *C. dubia* calls for starting test neonates within their first 24 h of life (ASTM International 2014). With *D. magna*, Traudt et al. (2016) showed that sensitivity of neonates to metals is highly variable even within their first 24 h postbirth. For example, 2‐ to 4‐h‐old *D. magna* neonates were approximately 2 times more sensitive to Zn than 0‐ to 4‐h‐olds, and 20‐ to 24‐h neonates were approximately 6 times more sensitive to Cd than 0‐ to 4‐h‐old neonates (Traudt et al. 2016). In the present study, because of the large number of treatments to be seeded (~140 beakers and 700 organisms), it was impractical to further constrain the age of tested neonates beyond their first 24 h after birth. Thus, developmental differences even within the conventionally acceptable 24‐h window for starting tests with daphnids could contribute considerable test variability. This apparently inherent variability presents challenges in test design and data interpretation. Data interpretations may need to be done on a relative basis, emphasizing comparisons within a study.

### Implications for aquatic life criteria acute averaging periods

One of the questions that could be asked of the test results is whether 1‐ or 24‐h averaging periods would provide the level of protection for fluctuating concentrations intended by the US national aquatic life criteria guidelines (Stephan et al. 1985). This question can be evaluated through the following simple calculations. The rainbow trout 96‐h LC50 is approximately 270 µg/L. If this LC50 was used as a 24‐h average, would it be protective of exposures shorter than 24 h? For the 8‐h exposure, a 24‐h average of 270 µg/L would allow a maximum 8‐h average concentration of 3 × 270 = 810 µg/L. Because the delayed‐mortality LC50 was 1450 µg/L, an 8‐h averaging period would be protective. Similarly, for the 3‐h exposure, the delayed‐mortality LC50 was 5600 µg/L compared to the maximum 2160 µg/L allowed by the 24‐h average. For the 1‐h exposure, the delayed‐mortality LC50 was >49 000 µg/L compared to an allowed 6480 µg/L. Therefore, for trout, the present study has demonstrated that a 24‐h averaging period would be appropriate even accounting for delayed mortality and that a 1‐h averaging period could be considered overly restrictive.

In contrast, for the *C. dubia* tested, the conclusions are different. The 48‐h LC50 was 37 µg/L (not including delayed mortality, which was minimal). The highest 1‐h concentration that would not exceed a 37 µg/L 24‐h concentration is 37 × 24 = 888 µg/L. But the 1 h delayed mortality LC50 was ~107 µg/L, which is far less than 888 µg/L allowed by a 24‐h average. Likewise, the maximum average concentration possible for a 3‐h exposure that did not exceed the 24‐h average concentration of 37 µg/L is 37 × 8 = 296 µg/L. Because the 3‐h delayed mortality was 100% at 114 µg/L, the lowest concentration tested, a 3‐h averaging period would be unprotective for *C. dubia* and Zn under the conditions tested.

The present results showed that short‐term episodic exposures of a sensitive invertebrate to Zn at moderate concentrations (1 to 2 times the acute aquatic life criteria) for 1‐ to 3‐h periods had little effect during the actual exposure periods but produced very high mortality within 36 h. Although rainbow trout exhibited fewer delayed mortalities than *C. dubia*, exposure periods of 8 h to Zn concentrations that caused few, if any, mortalities during the actual exposures were sufficient to cause 40 to 70% mortality to rainbow trout by 36 h. These results support the recommendations of Stephan et al. (1985) that when acute criteria are defined using the averaging period concept, the averaging period should be short enough to avoid allowing unacceptable effects to sensitive taxa, for which 1 h was recommended as an appropriate averaging time. The results are also consistent with work by Brent and Herricks (1998), who found that 2‐ to 4‐h exposures to Zn had no effect on daphnids during the exposure periods but that nearly 100% had died by 24 h.

## Supplemental Data

The Supplemental Data are available on the Wiley Online Library at https://doi.org/10.1002/etc.5131.

## Disclaimer

The views, findings, and any recommendations in the present study are those of the authors and are not intended to represent those of the USEPA. Any use of trade, firm, or product names is for descriptive purposes only and does not imply endorsement by the US government. The testing plan complied with institutional safety, biosecurity, animal welfare, quality assurance, and data management policies, details of which are available upon request. The authors have no competing interests to declare.

## Supporting information

This article includes online‐only Supplemental Data.

Supporting information.

Supporting information.

Supporting information.

Supporting information.

## Data Availability

Full data sets for the present study are available through the Supplemental Data and through a data repository at https://doi.org/10.5066/p9x4hh4t. Data, associated metadata, and calculation tools are also available from the corresponding author (cmebane@usgs.gov).

## References

[etc5131-bib-0001] Alsop D, Wood CM. 2011. Metal uptake and acute toxicity in zebrafish: Common mechanisms across multiple metals. Aquat Toxicol 105:385–393. 21820385 10.1016/j.aquatox.2011.07.010

[etc5131-bib-0002] Alsop DH, Wood CM. 2000. Kinetic analysis of zinc accumulation in the gills of juvenile rainbow trout: Effects of zinc acclimation and implications for biotic ligand modeling. Environ Toxicol Chem 19:1911–1918.

[etc5131-bib-0003] American Public Health Association, American Water Works Association, and Water Environment Federation . 2005. Method 3500‐Zn ZINC. In *Standard Methods for the Examination of Water and Wastewater*, 21st ed. Washington, DC, pp 3‐45–3‐52.

[etc5131-bib-0004] ASTM International . 2013. Standard guide for conducting three‐brood, renewal toxicity tests with *Ceriodaphnia dubia Annual Book of ASTM Standards*. Method E1295‐01 (reapproved 2013). In, Vol 11.04. Philadelphia, PA, USA.

[etc5131-bib-0005] ASTM International . 2014. Standard guide for conducting acute toxicity tests on test materials with fishes, macroinvertebrates, and amphibians. Method E729‐96 (reapproved 2014). In *Annual Book of ASTM Standards*, Vol 11.06. Philadelphia, PA, USA.

[etc5131-bib-0006] Balistrieri LS, Mebane CA, Schmidt TS. 2020. Time‐dependent accumulation of Cd, Co, Cu, Ni, and Zn in natural communities of mayfly and caddisfly larvae: Metal sensitivity, uptake pathways, and mixture toxicity. Sci Total Environ 732:139011. 32473394 10.1016/j.scitotenv.2020.139011

[etc5131-bib-0007] Balistrieri LS, Nimick DA, Mebane CA. 2012. Assessing time‐integrated dissolved concentrations and predicting toxicity of metals during diel cycling in streams. Sci Total Environ 425:155–168. 22481055 10.1016/j.scitotenv.2012.03.008

[etc5131-bib-0008] Berthouex PM, Fan R. 1986. Evaluation of treatment plant performance: Causes, frequency, and duration of upsets. J Water Pollut Control Fed 58:368–375.

[etc5131-bib-0009] Borgmann U, Norwood WP. 1995. Kinetics of excess (above background) copper and zinc in *Hyalella azteca* and their relationship to chronic toxicity. Can J Fish Aquat Sci 52:864–874.

[etc5131-bib-0010] Bownik A. 2020. Physiological endpoints in daphnid acute toxicity tests. Sci Total Environ 700:134400. 31689654 10.1016/j.scitotenv.2019.134400

[etc5131-bib-0011] Brent RN, Herricks EE. 1999. A method for the toxicity assessment of wet weather events. Water Res 33:2255–2264.

[etc5131-bib-0012] Brent RN, Herricks EE. 1998. Postexposure effects of brief cadmium, zinc, and phenol exposures on freshwater organisms. Environ Toxicol Chem 17:2091–2099.

[etc5131-bib-0013] Brix KV, Santore RC, DeForest DK, Tobiason S. 2010. Ecological risk assessment of zinc from stormwater runoff to an aquatic ecosystem. Sci Total Environ 408:1824–1832. 20035970 10.1016/j.scitotenv.2009.12.004

[etc5131-bib-0014] Butcher JB, Diamond JM, Bearr J, Latimer H, Klaine SJ, Hoang TC, Bowersox M. 2006. Toxicity models of pulsed copper exposure to *Pimephales promelas* and *Daphnia magna* . Environ Toxicol Chem 25:2541–2550. 16986811 10.1897/05-630r.1

[etc5131-bib-0015] Calfee RD, Little EE, Puglis HJ, Scott E, Brumbaugh WG, Mebane CA. 2014. Acute sensitivity of white sturgeon (*Acipenser transmontanus*) and rainbow trout (*Oncorhynchus mykiss*) to copper, cadmium, or zinc in water‐only laboratory exposures. Environ Toxicol Chem 33:2259–2272. 25043712 10.1002/etc.2684 PMC4278710

[etc5131-bib-0016] Corsi SR, Graczyk DJ, Geis SW, Booth NL, Richards KD. 2010. A fresh look at road salt: Aquatic toxicity and water‐quality impacts on local, regional, and national scales. Environ Sci Technol 44:7376–7382. 20806974 10.1021/es101333u PMC2947309

[etc5131-bib-0017] DeForest DK, Van, Genderen EJ. 2012. Application of U.S. EPA guidelines in a bioavailability‐based assessment of ambient water quality criteria for zinc in freshwater. Environ Toxicol Chem 31:1264–1272. 22447746 10.1002/etc.1810

[etc5131-bib-0018] Diamond JM, Klaine SJ, Butcher JB. 2006. Implications of pulsed chemical exposures for aquatic life criteria and wastewater permit limits. Environ Sci Technol 40:5132–5138. 16955918 10.1021/es0604358

[etc5131-bib-0019] Electric Power Research Institute . 2008. Development of an integrated episodic exposure and bioavailability model. Palo Alto, CA, USA. [cited 2018 March 1]. Available from: https://www.epri.com/research/products/1014065

[etc5131-bib-0020] Erickson RJ. 2007. Quantification of toxic effects for water concentration‐based aquatic life criteria, part A. EPA/600/R‐07/065. US Environmental Protection Agency, Duluth, MN. [cited 2020 April 23]. Available from: https://archive.epa.gov/med/med_archive_02/web/pdf/600r07065.pdf

[etc5131-bib-0021] Erickson RJ. 2015. Toxicity Relationship Analysis Program, Ver 1.30a. US Environmental Protection Agency, Duluth, MN. [cited 2021 March]. Available from: https://archive.epa.gov/med/med_archive_03/web/html/trap.html

[etc5131-bib-0022] Fisher DJ, Burton DT, Yonkos LT, Turley SD, Turley BS, Ziegler GP, Zillioux EJ. 1994. Acute and short‐term chronic effects of continuous and intermittent chlorination on *Mysidopsis bahia* and *Menidia beryllina* . Environ Toxicol Chem 13:1525–1534.

[etc5131-bib-0023] Gaillardet J, Viers J, Dupre B. 2007. Trace elements in river waters. In Drever JI, ed, Surface and Ground Water, Weathering, and Soils. Vol 5—Treatise on Geochemistry. Elsevier, Amsterdam, The Netherlands, pp 225–272.

[etc5131-bib-0024] Gergs A, Kulkarni D, Preuss TG. 2015. Body size–dependent toxicokinetics and toxicodynamics could explain intra‐ and interspecies variability in sensitivity. Environ Pollut 206:449–455. 26275729 10.1016/j.envpol.2015.07.045

[etc5131-bib-0025] Gordon AK, Mantel SK, Muller NWJ. 2012. Review of toxicological effects caused by episodic stressor exposure. Environ Toxicol Chem 31:1169–1174. 22447338 10.1002/etc.1781

[etc5131-bib-0026] Grosell M, Nielsen C, Bianchini A. 2002. Sodium turnover rate determines sensitivity to acute copper and silver exposure in freshwater animals. Comp Biochem Physiol C Toxicol Pharmacol 133:287–303. 12356534 10.1016/s1532-0456(02)00085-6

[etc5131-bib-0027] Hogstrand C. 2011. Zinc. In Wood CM, Farrell AP, Brauner CJ, eds, Homeostasis and Toxicology of Essential Metals. Vol 31A—Fish Physiology. Elsevier, Amsterdam, The Netherlands, 135–200.

[etc5131-bib-0028] Hogstrand C, Reid SM, Wood CM. 1995. Ca^2+^ versus Zn^2+^ transport in the gills of freshwater rainbow trout and the cost of adaptation to waterborne Zn^2+^ . J Exp Biol 198:337–348. 9317921 10.1242/jeb.198.2.337

[etc5131-bib-0029] Ivey CD, Besser JM, Ingersoll CG, Wang N, Rogers DC, Raimondo S, Bauer CR, Hammer EJ. 2017. Acute sensitivity of the vernal pool fairy shrimp, *Branchinecta lynchi* (Anostraca; Branchinectidae), and surrogate species to 10 chemicals. Environ Toxicol Chem 36:797–806. 28019706 10.1002/etc.3723

[etc5131-bib-0030] Ivey CD, Mebane CA. 2019. Acute and latent effects of zinc on two commonly tested species (*Ceriodaphnia dubia and Oncorhynchus mykiss*). US Geological Survey Data Release. [cited 2021 June 13]. Available from: 10.5066/p9x4hh4t

[etc5131-bib-0031] Kayhanian M, Stransky C, Bay S, Lau SL, Stenstrom MK. 2008. Toxicity of urban highway runoff with respect to storm duration. Sci Total Environ 389:386–406. 17920106 10.1016/j.scitotenv.2007.08.052

[etc5131-bib-0032] Lee H, Lau S‐L, Kayhanian M, Stenstrom MK. 2004. Seasonal first flush phenomenon of urban stormwater discharges. Water Res 38:4153–4163. 15491663 10.1016/j.watres.2004.07.012

[etc5131-bib-0033] Makepeace DK, Smith DW, Stanley SJ. 1995. Urban stormwater quality: Summary of contaminant data. Crit Rev Environ Sci Technol 25:93–139.

[etc5131-bib-0034] Mancini JL. 1983. A method for calculating effects, on aquatic organisms, of time varying concentrations. Water Res 17:1355–1362.

[etc5131-bib-0035] McRae NK, Gaw S, Glover CN. 2016. Mechanisms of zinc toxicity in the galaxiid fish. Galaxias maculatus. Comp Biochemy Physiol C Toxicol Pharmacol 179:184–190. 10.1016/j.cbpc.2015.10.010 26510681

[etc5131-bib-0036] Mebane CA. 2006. Cadmium risks to freshwater life: Derivation and validation of low‐effect criteria values using laboratory and field studies. Scientific investigation report 2006‐5245 (2010 rev.). US Geological Survey, Boise, ID.

[etc5131-bib-0037] Mebane CA, Chowdhury MJ, De Schamphelaere KAC, Lofts S, Paquin PR, Santore RC, Wood CM. 2020a. Metal bioavailability models: Current status, lessons learned, considerations for regulatory use, and the path forward. Environ Toxicol Chem 39:60–84. 31880840 10.1002/etc.4560

[etc5131-bib-0038] Mebane CA, Schmidt TS, Miller JL, Balistrieri LS. 2020b. Bioaccumulation and toxicity of cadmium, copper, nickel, and zinc to aquatic insect communities. Environ Toxicol Chem 39:812–833. 31916284 10.1002/etc.4663 PMC7154727

[etc5131-bib-0039] Meyer JS, Boese CJ, Morris JM. 2007. Use of the biotic ligand model to predict pulse‐exposure toxicity of copper to fathead minnows (*Pimephales promelas*). Aquat Toxicol 84:268–278. 17659358 10.1016/j.aquatox.2006.12.022

[etc5131-bib-0040] Muyssen BTA, Bossuyt BTA, Janssen CR. 2005. Inter‐ and intra‐species variation in acute zinc tolerance of field‐collected cladoceran populations. Chemosphere 61:1159–1167. 16263385 10.1016/j.chemosphere.2005.02.076

[etc5131-bib-0041] Muyssen BTA, De Schamphelaere KAC, Janssen CR. 2006. Mechanisms of chronic waterborne Zn toxicity in *Daphnia magna* . Aquat Toxicol 77:393–401. 16472524 10.1016/j.aquatox.2006.01.006

[etc5131-bib-0042] Muyssen BTA, Janssen CR. 2002. Accumulation and regulation of zinc in *Daphnia magna*: Links with homeostasis and toxicity. Arch Environ Contam Toxicol 43:0492–0496. 10.1007/s00244-002-1245-9 12399922

[etc5131-bib-0043] Nimick DA, Gammon JR, Parker SR. 2011. Diel biogeochemical processes and their effect on the aqueous chemistry of streams: A review. Chem Geol 283:3–17.

[etc5131-bib-0044] Playle RC. 1998. Modelling metal interactions at fish gills. Sci Total Environ 219:147–163.

[etc5131-bib-0045] Poteat MD, Buchwalter DB. 2014. Phylogeny and size differentially influence dissolved Cd and Zn bioaccumulation parameters among closely related aquatic insects. Environ Sci Technol 48:5274–5281. 24730589 10.1021/es501096a

[etc5131-bib-0046] Scholz NL, Myers MS, McCarthy SG, Labenia JS, McIntyre JK, Ylitalo GM, Rhodes LD, Laetz CA, Stehr CM, French BL, McMillan B, Wilson D, Reed L, Lynch KD, Damm S, Davis JW, Collier TK. 2011. Recurrent die‐offs of adult coho salmon returning to spawn in Puget Sound lowland urban streams. PLoS One 6:e28013. 22194802 10.1371/journal.pone.0028013 PMC3237429

[etc5131-bib-0047] Seegert GL, Brooks AS. 1978. The effects of intermittent chlorination on coho salmon, alewife, spottail shiner, and rainbow smelt. Trans Am Fish Soc 107:346–353.

[etc5131-bib-0048] Spry DJ, Wood CM. 1984. Acid–base, plasma ion and blood gas changes in rainbow trout during short term toxic zinc exposure. J Comp Physiol B 154:149–158.

[etc5131-bib-0049] Spry DJ, Wood CM. 1985. Ion flux rates, acid–base status, and blood gases in rainbow trout, *Salmo gairdneri*, exposed to toxic zinc in natural soft water. Can J Fish Aquat Sci 42:1332–1341.

[etc5131-bib-0050] Stephan CE, Mount DI, Hansen DJ, Gentile JH, Chapman GA, Brungs WA. 1985. Guidelines for deriving numerical national water quality criteria for the protection of aquatic organisms and their uses. EPA 822‐R‐85‐100, NTIS PB85 227049. US Environmental Protection Agency, Duluth, MN; Narragansett, RI; Corvallis, OR.

[etc5131-bib-0051] Todd AS, Brinkman SF, Wolf RE, Lamothe PJ, Smith KS, Ranville JF. 2009. Use of an enriched stable‐isotope approach to determine the gill‐zinc binding properties of juvenile rainbow trout (*Oncorhynchus mykiss*) during acute zinc exposures in hard and soft waters. Environ Toxicol Chem 28:1233–1243. 19132811 10.1897/08-252.1

[etc5131-bib-0052] Tomczyk N, Parr TB, Gray E, Iburg J, Capps K. 2018. Trophic strategies influence metal bioaccumulation in detritus‐based, aquatic food webs. Environ Sci Technol 52:11886–11894. 30226374 10.1021/acs.est.8b04009

[etc5131-bib-0053] Traudt EM, Ranville JF, Meyer JS. 2016. Effect of age on acute toxicity of cadmium, copper, nickel, and zinc in individual‐metal exposures to *Daphnia magna* neonates. Environ Toxicol Chem 36:113–119. 27225713 10.1002/etc.3507 PMC5764767

[etc5131-bib-0054] US Environmental Protection Agency . 1996. 1995 updates: Water quality criteria documents for the protection of aquatic life in ambient water. EPA 820‐B‐96‐001. Washington, DC.

[etc5131-bib-0055] US Environmental Protection Agency . 2001. Update of the ambient water quality criteria for cadmium. EPA/822/R‐01‐001. Washington, DC.

[etc5131-bib-0056] US Environmental Protection Agency . 2002a. Methods for measuring the acute toxicity of effluents and receiving waters to freshwater and marine organisms, 5th ed. EPA‐821‐R‐02‐012. Cincinnati, OH.

[etc5131-bib-0057] US Environmental Protection Agency . 2002b. Short‐term methods for estimating the chronic toxicity of effluents and receiving waters to freshwater organisms, 4th ed. EPA‐821‐R‐02‐013. Cincinnati, OH.

[etc5131-bib-0058] US Environmental Protection Agency . 2007. Aquatic life ambient freshwater quality criteria—Copper. EPA‐822‐R‐07‐001. Washington, DC.

[etc5131-bib-0059] US Environmental Protection Agency . 2013. Aquatic life ambient water quality criteria for ammonia—Freshwater. EPA 822‐R‐13‐001. Washington, DC.

[etc5131-bib-0060] US Environmental Protection Agency . 2016. Aquatic life ambient water quality criterion for cadmium. EPA‐820‐R‐16‐002. Washington, DC.

[etc5131-bib-0061] Veltman K, Huijbregts MAJ, Hendriks AJ. 2010. Integration of biotic ligand models (BLM) and bioaccumulation kinetics into a mechanistic framework for metal uptake in aquatic organisms. Environ Sci Technol 44:5022–5028. 20515030 10.1021/es903697c

[etc5131-bib-0062] Wang W‐X, Guan R. 2010. Subcellular distribution of zinc in *Daphnia magna* and implication for toxicity. Environ Toxicol Chem 29:1841–1848. 20821640 10.1002/etc.229

[etc5131-bib-0063] Zhao Y, Newman MC. 2004. Shortcomings of the laboratory‐derived median lethal concentration for predicting mortality in field populations: Exposure duration and latent mortality. Environ Toxicol Chem 23:2147–2153. 15378991 10.1897/03-557

[etc5131-bib-0064] Zhao Y, Newman MC. 2007. The theory underlying dose–response models influences predictions for intermittent exposures. Environ Toxicol Chem 26:543–547. 17373520 10.1897/06-398r.1

