# Supplementary material for: Direct and Delayed Mortality of Ceriodaphnia dubia and Rainbow Trout Following Time‐Varying Acute Exposures to Zinc
Source: Environ Toxicol Chem. 2021 Jul 20;40(9):2484–98. doi: 10.1002/etc.5131 (PMC8457064; doi:10.1002/etc.5131)
Supplement: Supplementary file 1 — Supporting information. [file ETC-40-2484-s001.pdf]

Guide to Supplemental Information to the article "*Direct and delayed mortality of Ceriodaphnia dubia and rainbow trout following time-varying acute exposures to zinc*"

Supporting online supplemental information for this article consists of 7 items published online under the "Supporting Information" section of the article's web page, in addition to a standalone data publication (Ivey and Mebane 2019). The items consist of the following:

S1 – Summary of test conditions for conducting acute tests with *Ceriodaphnia dubia*

S2 – Summary of test conditions for conducting acute tests with rainbow trout (*Oncorhynchus mykiss*)

S3 – Measured water quality parameters for *C. dubia* and *O. mykiss* tests.

S4 – Measured concentrations of major cations and anions and dissolved organic carbon in *C. dubia* and *O. mykiss* tests.

S5 – Measured Zn concentrations in *C. dubia* and *O. mykiss* tests.

S6 – Workbook which includes 10 worksheets with multiple tables each with survival counts and effects concentrations calculations for the 5 each toxicity tests with *C. dubia* and *O. mykiss.*

S7 – Additional information on chemical analytical methods and quality control results

Data Publication:

Ivey, C.D. and C.A. Mebane. 2019. Acute and latent effects of zinc on two commonly tested species (*Ceriodaphnia dubia* and *Oncorhynchus mykiss*). *U.S. Geological Survey Data Release*. https://doi.org/10.5066/P9X4HH4T.
