# Supplementary material for: Direct and Delayed Mortality of Ceriodaphnia dubia and Rainbow Trout Following Time‐Varying Acute Exposures to Zinc
Source: Environ Toxicol Chem. 2021 Jul 20;40(9):2484–98. doi: 10.1002/etc.5131 (PMC8457064; doi:10.1002/etc.5131)
Supplement: Supplementary file 4 — Supporting information. [file ETC-40-2484-s004.pdf]

Supplemental Information for the article "Direct and delayed mortality of *Ceriodaphnia dubia* and rainbow trout following time-varying acute exposures to zinc"

Additional information on chemical analytical methods and quality control results

Water samples for analyses of dissolved major ions and dissolved organic carbon (DOC) were collected from the control exposure chambers on Day 1 for C. dubia and Day 0 for rainbow trout. Samples were filtered through a 0.45 µm polyethersulfone [PES] membrane and preserved for analyses. Major ion samples for the cations calcium, magnesium, potassium, and sodium were preserved by acidification with high purity nitric acid, to result in a final concentration of 2 % HNO3 prior to analysis by inductively coupled plasma-mass spectrometry (ICP-MS). Major ion samples for the anions chloride and sulfate were refrigerated (4 °C) for up to 30 d prior to analyses by ion chromatography. The DOC samples were acidified to pH<2 with high purity sulfuric acid, refrigerated (4 °C), and analyzed within 28 d using a total organic carbon analyzer (Model TOC-L CSH, Shimadzu Scientific Instruments, Inc., Columbia, MD). Water samples for Zn analyses were generally collected from each exposure concentration at 0, 8, 24, and 48 h during the C. dubia test where Zn exposure lasted 48 h; and at 0, 8, 24, 48, and 96 h during the rainbow trout test where Zn exposure lasted 96 h. Treatments where Zn exposures were relatively shorter tracked similar water collection time points, with latest collection points being limited by the duration of the individual Zn exposures

Two methods of analysis were used to measure dissolved Zn concentrations in the exposure chambers over time. In the first analytical method, colorimetric analyses of Zn were performed using a visible spectrophotometer (Model HI801, Hanna Instrument, Smithfield, RI) where Zn concentrations in filtered test water samples (filtered with 0.45 µm PES) were determined using the zincon standard method (APHA 2005). Briefly, cyanide was used to complex Zn and other metals in solution; and cyclohexanone was then added to selectively release Zn from the cyanide complex. The newly-liberated Zn ions complexed with the zincon indicator in a pH 9 buffered solution, causing a measurable color change at 620 nm that was proportional to the Zn ion concentration. The linear range of the spectrophotometer was 0-3.00 mg Zn/L, the baseline resolution was 0.01 mg Zn/L, and the quantification limit (LOQ) was 0.03 mg Zn/L. Water samples were collected and measured by colorimetric analyses at the beginning and the end of all exposures (except at the end of the 2 short 1- and 3- h exposures) and the end of the recovery times (Table 1). In the second analytical method, concentrations of dissolved Zn (0.45 µm PES) in test waters were measured using inductively coupled plasma-mass spectrometry (ICP-MS;); samples for ICP-MS analyses were collected at 24-h for *C. dubia* and at 0-, 48-, and 96-h for rainbow trout. The Zn samples for ICP-MS analyses were preserved by acidification with high purity nitric acid, to result in a final acid concentration of 2 % HNO_3.

The inductively coupled plasma-mass spectrometry (ICP-MS) method for Zn and the major cations was similar to USEPA 6020B (USEPA 2014). The ICP-MS (NexION 2000; PerkinElmer, Illinois, USA) was equipped with an autosampler and auto-dilutor, and a minimum of three external National Institute of Standards and Technology (NIST)-traceable calibration standards plus a calibration blank were used to calibrate the instrument response. Established laboratory quality assurance/quality control

(QA/QC) procedures and sample types (e.g., second source calibration verification standards, analysis spikes and duplicates, and the use of certified reference materials or laboratory control samples) were used to verify instrument performance, accuracy, and precision throughout the ICP-MS analyses. The QC results for the major cations indicated that the results were generally in-control; recoveries of the cations from certified reference materials (CRMs) and laboratory control samples (LCSs) were 96-130 %. Relative percent differences (RPD) between analysis duplicates were 0-2 % RPD, analysis spikes had 96-104 % recoveries, and percent differences between undiluted and 5x-diluted samples were 0-18 %. The reporting limit for each major cation was 0.1 mg/L. Recoveries of Zn from CRMs were 103-108 %; Zn recovery from an interference check solution was 83 %. Analysis duplicates for Zn had 2.1 % RPD, and analysis spikes had 95 % recovery. The percent difference between an undiluted and 5x-diluted sample was 3.2 %. The estimated limit of detection (LOD) for Zn by ICP-MS was 1 µg/L, and the estimated LOQ was 10 µg/L.

Chloride and sulfate were analyzed by anion chromatography with suppressed conductivity detection (ICS-1100; Dionex, Sunnyvale, CA, USA) using a method similar to USEPA 9056A. The ICS-1100 system was equipped with a Dionex Ionpac AS22 anion exchange guard and analytical columns; and a 4.5 mM sodium carbonate/1.4 mM sodium bicarbonate mobile phase was used to elute the target anions within 13 min. A minimum of three NIST-traceable external calibration standards were used to calibrate the instrument response; and standard QA/QC measures were used to verify instrument performance throughout the analyses. The reporting limit for chloride was 0.3 mg/L, and the reporting limit for sulfate was 1.5 mg/L. Analyte recoveries from laboratory control samples were 90-102 %. Relative percent differences between analysis duplicates were 0 % for sulfate and 0-9.5 % for chloride. Recoveries of analysis spikes were 116-133 % for chloride and 106-113 % for sulfate.

Dissolved organic carbon (DOC) was measured as non-purgeable organic carbon (NPOC) by high temperature catalytic oxidation-nondispersive infrared spectroscopy using a total organic carbon analyzer (Model TOC-L CSH, Shimadzu Scientific Instruments, Inc., Columbia, MD). The method was similar to USEPA 415.3 (Potter and Wimsatt 2009). The samples were air sparged to remove inorganic carbon species prior to the NPOC measurements. The RPD between analysis duplicates was 0 %, and an analysis spike recovery from a test sample was 110 %. The LOD was 0.4 mg/L DOC, and the limit of identification was 2 mg/L.

References

APHA. 2005. Method 3500-Zn ZINC. Pages 3-45 to 3-52 *in Standard methods for the examination of water and wastewater*, 21st edition. American Public Health Association, American Water Works Association, and Water Environment Federation, Washington, D.C. .

Potter, B.B. and J.C. Wimsatt. 2009. Method 415.3 Determination of total organic carbon and specific UV absorbance at 254 nm in source water and drinking water. EPA/600/R-09/122, U.S. Environmental Protection Agency, Cincinatti, Ohio, Cincinatti, Ohio.

USEPA. 2014. Method 6020B: Inductively coupled plasma-mass spectrometry (SW-846). . Washington (DC): US Environmental Protection Agency. .
